# Molecular Characterization of Syndromic Hearing Loss in North African Moroccan Families

**DOI:** 10.3390/biom16050619

**Published:** 2026-04-22

**Authors:** Khawla El Fizazi, Amama Ghaffar, Laila Bouguenouch, Irum Badshah Saleem, Karim Ouldim, Zubair M. Ahmed, Mohammed Ridal, Saima Riazuddin

**Affiliations:** 1 Laboratory of Biomedical and Translational Research, Faculty of Medicine, Pharmacy and Dentistry, Sidi Mohamed Ben Abdellah University, Fez 30000, Morocco; khawla.elfizazi@usmba.ac.ma (K.E.F.); lbouguenouch@yahoo.com (L.B.); karim.ouldim@irc.ma (K.O.); 2 Unit of Medical Genetics and Oncogenetics, Hassan II University Hospital, Fez 30050, Morocco; 3 Department of Otorhinolaryngology-Head & Neck Surgery, University of Maryland, Baltimore, MD 21201, USA; aghaffar@som.umaryland.edu (A.G.); badshah.saleem85@gmail.com (I.B.S.); zmahmed@som.umaryland.edu (Z.M.A.); 4 Department of Biochemistry and Molecular Biology, University of Maryland, Baltimore, MD 21201, USA; 5 Marlene & Stewart Greenebaum Comprehensive Cancer Center, University of Maryland, Baltimore, MD 21201, USA; 6 Department of Otorhinolaryngology, Hassan II University Hospital, Fez 30050, Morocco; mohammed.ridal@usmba.ac.ma

**Keywords:** syndromic hearing loss (SHL), exome sequencing (ES), North African population, genetic heterogeneity

## Abstract

Hearing loss (HL) is a common sensory disorder, with syndromic forms accounting for ~30% of genetic cases. Due to phenotypic and genetic heterogeneity, accurate diagnosis remains challenging. Exome sequencing (ES) offers a powerful tool to uncover the underlying genetic causes. This study aimed to investigate the genetic basis of syndromic hearing loss (SHL) in North African Moroccan patients through ES. Seven individuals with suspected SHL were recruited from Hassan II University Hospital, Fez. After clinical and audiological assessments, ES was performed to identify causal genetic variants. Two of the participating individuals had Usher syndrome, and one each had Cornelia de Lange syndrome, Wolfram syndrome, Jervell and Lange-Nielsen syndrome, CHARGE syndrome, and Waardenburg syndrome. The causes of all these syndromes were determined, with pathogenic variants in *MYO7A*, *USH1G*, *SMC1A*, *WFS1*, *KCNQ1*, *CHD7*, and *MITF*. Across the combined cohort of reported Moroccan SHL cases, variants in *CHD7* and *MYO7A* were among the most frequently observed, while *USH1G* and *MITF* variants were rare. This study enhances the understanding of SHL in North Africa, revealing a high level of locus and allelic heterogeneity. Examining disparate populations yields novel insights into the etiology of SHL, which can subsequently enhance genetic diagnosis and tailored management strategies.

## 1. Introduction

Hearing loss (HL) is one of the most common sensory disorders worldwide, affecting approximately 2–3 in every 1000 newborns [1]. HL can be acquired, e.g., due to infectious diseases, physical injuries, or ototoxic drugs, or attributed to genetic factors, which account for 50–70% of cases [2]. Among the genetic forms, HL is classified as either non-syndromic (NSHL) when it occurs in isolation or syndromic (SHL), when accompanied by additional clinical features affecting other organs or systems such as the eyes, kidneys, skin, etc.

SHL accounts for approximately 30% of genetic HL cases [3]. So far, more than 400 genetic syndromes have been reported to include hearing loss as a feature [4], with the most prevalent syndromes in clinical settings being Pendred syndrome, Alport syndrome, Usher syndrome, Waardenburg syndrome, Branchio-oto-renal syndrome, and CHARGE syndrome [5]. While diagnosis of some syndromic forms can be established based on characteristic physical findings, others present significant diagnostic challenges due to overlapping phenotypes, variable expressivity, and progressive clinical manifestations with age. In addition to phenotypic diversity, genetic and allelic heterogeneity are major challenges in the diagnosis of SHL. Over two hundred genes have been linked to SHL, and many other genes are still to be discovered. Moreover, different variants within the same gene can lead to distinct clinical manifestations [6], further complicating accurate clinical diagnosis and effective patient management.

In this context, advanced genetic testing has become a critical tool for elucidating the underlying causes of Mendelian disorders, including SHL, facilitating early diagnosis, accurate clinical management, and informed genetic counseling. Exome sequencing (ES), in particular, has transformed the diagnostic approach to rare genetic conditions, as it enables the analysis of all the coding regions across the genome, including the identification of novel disease-associated genes [7].

While extensive progress has been made in identifying the genetic basis of SHL globally, data from North African populations, including Morocco, remain limited. Given the high rates of consanguinity and unique genetic diversity in this region, studying such populations offers an opportunity to expand the current understanding of the genetic and phenotypic spectrum of syndromic HL. Here, we performed ES on Moroccan patients with varying degrees of clinical suspicion of SHL.

## 2. Materials and Methods

### 2.1. Patient Recruitment and Clinical Assessment

Patients with possible syndromic hearing loss were recruited from the medical genetics unit and the otorhinolaryngology department at Hassan II University Hospital in Fez. The families were from different regions of Morocco. All participants underwent a comprehensive evaluation process, including a clinical ENT (Ear–Nose–Throat) examination, audiologic testing, and medical assessments, to accurately characterize clinical co-morbidities. Written informed consent was obtained from all participants or legal guardians in cases of minors. This study was approved by the Ethics Committee of Hassan II University Hospital, Fez, Morocco, and assigned Approval No. 19/02.

### 2.2. Exome Sequencing and Bioinformatic Analysis

Blood samples were obtained from all participants. Genomic DNA was extracted using a Maxwell RSC 48 Instrument(Promega Corporation, Madison, WI, USA) with a Maxwell RSC Blood DNA Kit, and DNA concentration (ng/μL) was assessed using a Qubit 3 fluorometer (Thermo Fisher Scientific, Waltham, MA, USA). De-identified DNA samples were shared with investigators at the University of Maryland, Baltimore (UMB), USA, for exome sequencing. At UMB, exome enrichment was conducted with an Agilent SureSelect Human Expanded All Exon V7 kit (Agilent Technologies, Santa Clara, CA, USA). Sequencing was performed on an Illumina HiSeq 4000 platform (Illumina, San Diego, CA, USA), producing 150 bp paired-end reads at an average of 100-fold coverage per sample.

Resulting genetic data was analyzed as described previously [8]. Briefly, sequencing reads were aligned to the GRCh37 human reference genome using the Burrows–Wheeler Aligner v0.7.17. The preprocessing steps involved removing duplicates with Picard v2.27.5 and SAMToolsv1.16.1, followed by base quality recalibration and realignment with the Genome Analysis Toolkit (GATK). Variant calling was carried out using both GATK HaplotypeCallerv4.3.0.0 and FreeBayesv1.3.6, generating variant call format (VCF) files.

For variant prioritization, Exomiser v13.0.0 was utilized to identify potential disease-associated variants. Only high-quality variants were considered based on predefined thresholds for read depth (≥4) and variant quality score (≥20). Protein-altering variants, including missense, nonsense, frameshift, and splice-site variants, were prioritized. Variant interpretation was performed under both autosomal recessive and autosomal dominant inheritance models, taking into account the syndromic presentation, with particular consideration of homozygous, compound heterozygous, and heterozygous variants. Exomiser incorporated these criteria, along with phenotype-based filtering through Human Phenotype Ontology (HPO) terms, inheritance patterns, and a frequency threshold of ≥0.01.

### 2.3. In Silico Analysis of Identified Genetic Variants

The potential impact of the selected variants on the encoded proteins was evaluated using the Combined Annotation Dependent Depletion (CADD) score (https://cadd.gs.washington.edu/score, accessed on 2 March 2026), AlphaMissense (https://alphamissense.hegelab.org/, accessed on 2 March 2026), MutationTaster (http://www.mutationtaster.org/, accessed on 2 March 2026), M-CAP (M-CAP: Mendelian Clinically Applicable Pathogenicity Score, Bejerano Lab, Stanford University, http://bejerano.stanford.edu/MCAP/, accessed on 2 March 2026) and phyloP100way (https://genome.ucsc.edu/cgi-bin/hgTrackUi?db=hg37&g=phyloP100way, accessed on 2 March 2026). Allele frequencies were assessed through gnomAD (https://gnomad.broadinstitute.org/, accessed on 2 March 2026), and all variants were classified based on the variant interpretation guidelines established by the American College of Medical Genetics and Genomics (ACMG). Then, 3D protein modeling was done using PyMOL(TM) 3.1.7.2 by obtaining PDB structures of SMC1A and CDH7 from I-Tasser to further assess the impact on protein structure. Lastly, splice-site variant impacts were predicted using the SpliceAI v1.3.1 tool (SpliceAI Lookup).

## 3. Results

This study included seven unrelated patients with suspected SHL, all of whom underwent exome sequencing to establish a molecular diagnosis. In four patients, the clinical presentation aligned with well-defined syndromes, while in the remaining patients, clinical manifestations did not clearly indicate a particular syndrome prior to genetic testing. The genetic findings are summarized in Table 1.

### 3.1. Case-by-Case Findings

#### 3.1.1. Patients 1 (Fam12) and 2 (Fam74) Have Usher Syndrome Type I

Patient 1 was a 15-year-old male born to consanguineous parents, with no family history of the deafness. The patient exhibited congenital hearing deficits, and audiometric evaluations confirmed profound sensorineural hearing loss. Motor development was delayed, with independent walking achieved at 36 months. Over time, he developed visual difficulties, and electrophysiological findings were consistent with rod–cone dystrophy characteristic of retinitis pigmentosa. These clinical features align with the classic presentation of Usher syndrome type 1 (USH1). ES data analysis of this patient revealed the presence of a homozygous pathogenic deletion of six coding nucleotides, i.e., c.653_658del within the *MYO7A* gene, which is known to cause USH1B. The identified variant is predicted to cause in-frame deletion of two amino acids, i.e., p.(Ile219_His220del) from the motor domain of the resulting MyosinVIIA protein (Table 1 and Table 2).

#### 3.1.2. Patient 2, a 10-Years-Old Female Born to Consanguineous Parents, Presented with Congenital Profound HL at the First Clinical Examination

ES analysis revealed a homozygous transition variant, c.742C>T in the *USH1G* gene, also known to cause the USH1 phenotype. The identified variant is predicted to introduce a premature stop codon at amino acid position 248 (p.Gln248*), resulting in a truncated protein. These findings support a diagnosis of Usher syndrome and led to referral for further clinical and visual functional assessments. At the time of the first clinical evaluation, the patient did not exhibit visual difficulties or other syndromic features. Following the genetic results, which were completed subsequently, she began reporting visual difficulties and was referred for comprehensive ophthalmologic assessment, with results pending.

#### 3.1.3. Patient 3 (Fam23) Exhibits Clinical Features of Cornelia De Lange Syndrome

**Figure 1:**
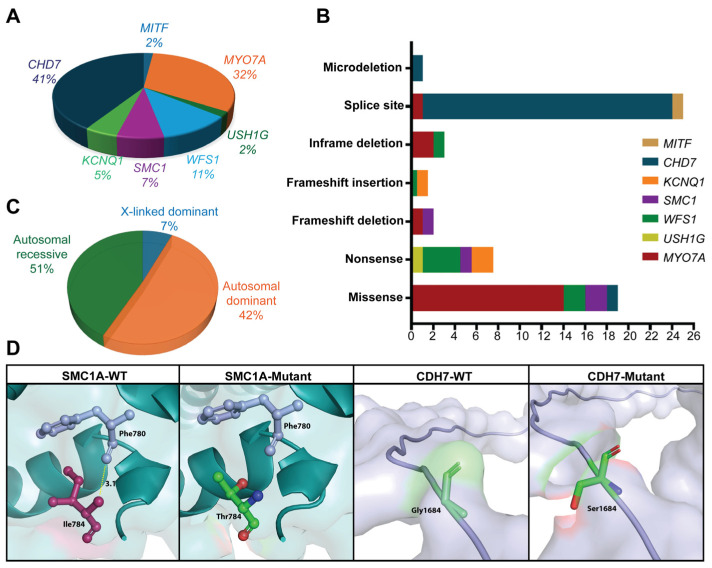
(**A**) Pie chart describing the overall percentages of prevalent genes identified in this study, along with their overall prevalence ratios in the Moroccan population *n* = 59. (**B**) Bar graph showing the prevalence of overall variants reported previously and identified in this study. (**C**) Modes of inheritance observed in Moroccan patients with syndromic hearing loss. (**D**) The 3D protein modeling of SMC1A and CHD7 missense variants: SMC1A-WT shows residues p.Ile784 (burgundy) and p.Phe780 (light purple) within an α-helical coiled-coil region; the dotted yellow line indicates a hydrogen bond between both amino acids. The SMC1A-Mutant displays p.(Ile784Thr) substitution and loss of hydrogen bond with p.Phe780. CHD7-WT shows residue p.Gly1684 (Green) in the loop region. The CHD7-Mutant displays the p.Gly1684Ser substitution.

Two 4-year-old monozygotic female twins born to non-consanguineous and healthy parents presented with hearing loss, feeding difficulties, delayed walking, intellectual disability, and the characteristic facial dysmorphia features associated with Cornelia de Lange syndrome. ES analysis revealed the presence of a heterozygous pathogenic variant, c.2351T>C within the *SMC1A* gene, an X-linked gene associated with Cornelia de Lange syndrome 2. The c.2351T>C variant is a dominant missense variant that leads to the substitution of isoleucine with threonine at position 784 [p.(Ile784Thr)] located within an α-helical coiled-coil region and is predicted to destabilize local protein structure. Further, multiple in silico pathogenicity prediction tools consistently classified this variant as pathogenic, supporting a deleterious functional effect (see Table 3). The 3D structural protein modeling demonstrated that substitution of p.Ile784 with p.Thr784 disrupts local hydrophobic interactions within the α-helical region, resulting in loss of the stabilizing interaction with the neighboring residue, p.Phe780 (Figure 1D).

#### 3.1.4. Patient 4 (Fam3) Has Wolfram Syndrome

A 17-year-old female patient exhibited a complex set of medical conditions, including type 1 diabetes, optic atrophy, open-angle glaucoma, polyuria–polydipsia syndrome, and mild mixed hearing loss that appeared progressively. This clinical presentation was suggestive of Wolfram syndrome; however, for confirmation, we performed ES. Resulting genomic data analysis revealed a homozygous pathogenic variant, c.1235_1237del, within the *WFS1*, a gene known to cause Wolfram syndrome. The identified recessive variant is predicted to cause an in-frame deletion of three base pairs, leading to the loss of a phenylalanine residue at position 414 (p.(Phe414del)).

#### 3.1.5. Patient 5 (Fam31) Has Jervell and Lange-Nielsen Syndrome

In a consanguineous family, two female siblings, aged 7 and 3 years, presented with congenital profound hearing loss and long QT syndrome, a diagnosis consistent with Jervell and Lange-Nielsen Syndrome (JLNS). The older sibling had passed away due to a sudden cardiac arrest. ES was performed on the younger sibling and revealed a homozygous c.915G>A variant within the *KCNQ1* gene, which is predicted to introduce a premature stop codon, p.(Trp305*) in the encoded protein.

#### 3.1.6. Patient 6 (Fam15) Genetic Analysis Confirmed CHARGE Syndrome

One 16-year-old female patient presented with asymmetrical sensorineural deafness characterized by mild hearing loss in the right ear and profound hearing loss in the left ear, which progressed over time. She also exhibited several associated phenotypes, including Mondini malformation, aplasia of the vestibulocochlear nerve and semicircular canal, hypoplasia of the cochlea, and outer-ear malformation. The Mondini triad is considered incomplete, as an enlarged vestibular aqueduct (EVA) was not identified or reported upon imaging. Additionally, she had growth retardation and mild intellectual disability. No other symptoms were noted, and the overall clinical presentation did not align with any well-defined syndrome. Genetic analysis of the ES data effectively identified a heterozygous previously reported pathogenic variant, c.5050G>A (p.(Gly1684Ser)), within the *CHD7* gene, which is known to cause CHARGE syndrome. These genetic findings provided a definitive diagnosis, improving the understanding of the patient’s condition, and guiding subsequent clinical management. Multiple in silico pathogenicity prediction tools consistently classified this variant as pathogenic (Table 3), and structural analysis demonstrated that substitution of the highly flexible p.Gly with p.Ser at residue position 1684 reduces local backbone flexibility and is predicted to alter protein conformation, despite no observable changes in hydrogen-bond interactions (Figure 1D).

#### 3.1.7. Waardenburg Syndrome Was Identified in Patient 7 (Fam20)

Within our cohort, a 4-year-old male patient presented with congenital profound hearing loss and very pale blue eyes, neither of which was evident within his family. Although these clinical symptoms were suggestive of Waardenburg syndrome, the absence of skin or hair pigmentary anomalies made clinical diagnosis uncertain. Our subsequent analysis of ES data effectively identified a heterozygous pathogenic splice-site variant, c.1031+1G>A, within the *MITF* gene, which is known to be implicated in the development of Waardenburg syndrome type 2. In silico splicing analyses for this variant predicted a strong disruption of the canonical splice donor site. Both splice site-specific and Pangolin analyses indicated that splice donor loss is the dominant effect, with a markedly higher splice-loss score of 0.82 as compared to a cryptic splice-gain potential of 0.34, supporting aberrant splicing as the primary molecular consequence (Table 3) in this patient.

Next, we also compared the findings of our study with all the reported genetic cases of SHL in the Moroccan population (see Table 2). Taken together, variants in the *CHD7* and *MYO7A* genes are the most frequent cause of SHL, accounting for approximately 41% and 32% of the reported alleles, while variants *USH1G* and *MITF* were rarely observed (see Figure 1A–C).

## 4. Discussion

Syndromic hearing loss (SHL) is an extremely heterogeneous condition, both clinically and genetically, making diagnosis particularly challenging. Clinically, it encompasses a broad range of syndromes, with over 400 identified, affecting multiple organ systems and often with overlapping features, which further challenge differential diagnosis [4]. Genetically, SHL is associated with a wide spectrum of genes and alleles across different inheritance patterns, with autosomal dominant being the most prevalent inheritance pattern [25]. This complexity underscores the need for advanced genetic testing approaches like exome sequencing (ES) to aid in accurate diagnosis and management.

There has only been limited investigation of SHL in the North African population. This study investigated the underlying genetic cause of SHL in seven North African families enrolled from Morocco. Of these seven participants, four had clinical features suggestive of a known syndrome, while in the remaining three subjects, differential diagnosis was required for further clinical management. Subsequently, we performed a comprehensive genetic analysis of these patients through ES to identify causative variants and inform clinical diagnosis. Known genes were identified as the underlying cause of SHL for all these cases, providing a confirmed diagnosis. In our cohort, four of the seven diagnosed cases displayed autosomal recessive inheritance, while the remaining three had heterozygous variants.

Variants in USH1-causing genes *MYO7A* and *USH1G* were identified in two patients. USH1, the most severe form of the disorder, is characterized by congenital bilateral SNHL, vestibular dysfunction, and prepubertal onset of retinitis pigmentosa [26,27]. The first patient presented with congenital profound hearing loss; delayed motor development, often reflecting vestibular dysfunction; and progressive visual impairment consistent with retinitis pigmentosa, leading to a strong suspicion of Usher syndrome, which was genetically confirmed through the identification of the c.653_658del (p.(Ile219_His220del)) variant in *MYO7A*. Pathogenic variants in the *MYO7A* gene can lead to either Usher syndrome or non-syndromic hearing loss (DFNB), depending on the specific variant’s type, position, and effect on protein function [28,29]. However, the p.(Ile219_His220del) variant has been previously reported in individuals with USH1 [9], thereby supporting the clinical diagnosis in our case.

The second patient initially presented with isolated congenital profound HL. However, during follow-up, she reported visual difficulties that had not yet been clinically investigated, and no syndromic diagnosis had been established. We identified a homozygous truncating allele, c.742C>T:p.(Gln248*), in the *USH1G* gene. The SANS protein, encoded by *USH1G*, as an adapter protein, facilitates USH interactome organization in inner-ear hair cells and retinal photoreceptor cells [30]. Notably, pathogenic variants in the *USH1G* gene are extremely rare [31], and to the best of our knowledge, patient 2 is the first report of the *USH1G* variant in the Moroccan population (Table 2).

Cornelia de Lange syndrome (CdLS) was diagnosed in monozygotic twins from our cohort, marked by variable features including distinctive facial traits, intellectual disability, growth issues, limb anomalies, and hearing loss [32]. They carry an *SMC1A* c.2351T>C:p.(Ile784Thr) variant in the hinge domain, which is crucial for cohesin-complex function in chromatid cohesion and gene regulation [33]. CdLS arises from variants in cohesin pathway genes, with *SMC1A* variants accounting for about 5% of cases. This hinge-domain variant causes overlapping CdLS and Rett-like symptoms by disrupting cohesin dynamics [34]. Prior studies reported that Moroccan patients with heterozygous *SMC1A* variants p.(Lys1063Argfs*149) in coiled coil domain 2 and nonsense p.(Gln222*) in coiled coil domain 1 present Rett-like and classic CdLS phenotypes, respectively [21]. Despite affecting different domains, all variants ultimately impact cohesin function to different extents, likely explaining the broad clinical variability observed in intellectual disability, growth, limb, and hearing impairments in individuals inheriting these variants. To further understand the molecular impact of the SMC1A p.(Ile784Thr) variant, in silico pathogenicity prediction and 3D protein modeling were done. The missense change was predicted to be damaging by multiple tools (Table 1 and Table 3). The 3D protein structural analysis showed the affected residue within an α-helical coiled-coil region adjacent to the hinge domain, where p.Ile784 makes 3.1 Å bond with p.Phe780 (Figure 1). Substitution of p.Ile with p.Thr, a less hydrophobic residue, resulted in the loss of the stabilizing interaction with the neighboring residue, p.Phe780, which is predicted to cause changes in protein folding. This further explains how missense changes in conserved structural regions of SMC1A can impair cohesin function and contribute to phenotypic variability in CdLS.

Wolfram syndrome 1 (WFS1) is a rare autosomal recessive neurodegenerative condition distinguished by its hallmark features of insulin-dependent diabetes mellitus, optic atrophy, diabetes insipidus, and hearing loss [35], with an estimated prevalence of 1 in 770,000 [35]. Additional features include urodynamics disorders, ataxia, and a spectrum of neurological and psychiatric abnormalities [35,36]. WFS1 results from biallelic pathogenic variants in the *WFS1* gene, which encodes Wolframin, a known regulator of the ER stress signaling network and cell apoptosis [37]. In our patient, a homozygous in-frame deletion, c.1235_1237del:p.(Phe414del), was identified, which has previously validated as pathogenic through functional studies [38].

In our cohort, we also identified two siblings inheriting Jervell and Lange-Nielsen syndrome (JLNS), a rare autosomal recessive disorder defined by congenital sensorineural hearing loss, a prolonged QT interval, and life-threatening arrhythmias [39]. Primarily, variants in *KCNQ1* account for 90% of JLNS cases, whereas *KCNE1* accounts for only 10%—both encoding proteins essential for potassium transport channel function in the heart and ear [40]. In our family, a rare homozygous known pathogenic nonsense variant in *KCNQ1* (p.Trp305*) was identified. Given the importance of loss-of-function variants in *KCNQ1* underlying JLNS, a condition with early onset and high risk of sudden death, it should be noted that heterozygous carriers of these variants, although not affected by the full syndrome, may exhibit mild or borderline QT-interval prolongation, reflecting variable cardiac expression. Our genetic findings highlight the need for careful clinical management, with particular attention to cardiac function in the surviving proband. Interestingly, in two cases with unclear clinical signs, our genetic screening results enabled diagnosis. Patient 7 (Fam3) had profound hearing loss and light-blue irises without typical pigmentation anomalies, making a diagnosis of Waardenburg syndrome type 2 (WS2) uncertain. Genetic testing revealed a heterozygous splice-site *MITF* variant (c.1031+1G>A), supporting WS2 diagnosis, an autosomal dominant disorder marked mainly by sensorineural hearing loss and pigmentary anomalies, but lacking dystopia canthorum, a phenotype commonly observed in WS1 [41]. The *MITF* encodes a transcription factor critical for melanocyte development and pigment production [42]. Although previously reported in other populations [15], patient 7 represents the first reported case of the *MITF* c.1031+1G>A variant in the Moroccan population. Splicing prediction analyses revealed that this variant likely disrupts the normal splice donor site. The donor loss score (0.99) and Pangolin splice-loss score (0.82) were much higher than the predicted splice-gain score (0.34), indicating that loss of normal splicing is the main effect of this variant. This suggests that the variant is likely to cause abnormal *MITF* transcripts rather than proper splicing. Such splicing disruption provides a clear explanation for the patient’s hearing loss and supports the pathogenic role of this variant in Waardenburg syndrome type 2. Similarly, patient 6 (Fam15) presented with asymmetric hearing loss, ear malformations, and mild developmental delay. Genetic analysis revealed a pathogenic *CHD7* variant (c.5050G>A). *CHD7* encodes an ATP-dependent chromatin remodeling protein essential for regulating gene expression during embryonic development and the formation of multiple organs, including the brain, inner ear, and craniofacial structures [43]. Variants in *CHD7* are known to cause CHARGE syndrome, characterized by coloboma, heart defects, choanal atresia, growth retardation, genital anomalies, and ear abnormalities including hearing loss [43]. In silico pathogenicity analyses confirm the pathogenic effect of the p.(Gly1684Ser) variant (Table 3). However, 3D protein modeling did not show any hydrogen bond impact, although the replacement of p.Gly, a highly flexible residue, with p.Ser at position 1684 is suspected to introduce steric constraints that can alter protein conformation.

In summary, although our study represents a small subset of the population, it underscores the critical role of genetic testing in diagnosing syndromic hearing loss within the Moroccan population, which is characterized by marked clinical and genetic heterogeneity. These findings expand current knowledge of syndromic hearing-loss genetics in Morocco and emphasize the importance of early genomic diagnosis for presymptomatic assessment and tailored management. Our results advocate for the integration of genetic testing, via exome sequencing, into routine clinical practice to improve diagnostic accuracy, guide multidisciplinary care, and enhance genetic counseling, especially for patients with incomplete or evolving syndromic features.

## Figures and Tables

**Table 1:** Summary of genetic variants identified in affected probands.

Patient ID	Gene	Transcript	Nucleotide Change	Amino Acid Change	Variation	Exon	Inheritance	Genotype	Variant Classification	Syndrome/Phenotype	REF	MIM Phenotype
**Fam12**	*MYO7A*	NM_000260.4	c.653_658del	p.(Ile219_His220del)	Inframe deletion	7	Autosomal recessive	Homozygous	Likely Pathogenic: [PM1, PM2, PM4, PP4, PP5]	Usher syndrome type 1B	[9]	276900
**Fam74**	*USH1G*	NM_173477.5	c.742C>T	p.(Gln248*)	Nonsense	2	Autosomal recessive	Homozygous	Pathogenic: [PVS1, PM2 PP4, PP5]	Usher syndrome type 1G	[10]	606943
**Fam3**	*WFS1*	NM_006005.3	c.1235_1237del	p.(Phe414del)	Inframe deletion	8	Autosomal recessive	Homozygous	Likely Pathogenic: [PM4, PM2, PP5, PP4]	Wolfram syndrome 1	[11]	222300
**Fam23**	*SMC1A*	NM_006306.4	c.2351T>C	p.(Ile784Thr)	Missense	15	X-linked-dominant	Heterozygous	Pathogenic: [PP5, PM1, PP3, PM2]	Cornelia de lange syndrome 2	[12]	300590
**Fam31**	*KCNQ1*	NM_000218.3	c.915G>A	p.(Trp305*)	Nonsense	6	Autosomal recessive	Homozygous	Pathogenic: [PVS1, PM2, PP5]	Jervell and Lange-Nielson syndrome	[13]	220400
**Fam15**	*CHD7*	NM_017780.4	c.5050G>A	p.(Gly1684Ser)	Missense	22	Autosomal dominant	Heterozygous	Pathogenic: [PP3, PP5, PM5, PS3, PM2]	CHARGE Syndrome	[14]	214800
**Fam20**	*MITF*	NM_001354604.2	c.1031+1G>A	p.?	Splice site variant	8	Autosomal dominant	Heterozygous	Pathogenic: [PVS1, PP5, PM2]	Waardenburg syndrome, type 2A	[15]	193510

**Table 2:** Identified genes, reported variants, and their details in Moroccan population.

Gene	Variant	Protein Change	Mutation Type	MOI	Associated Phenotype	Number of Patients	Domain	Patient Origin	Reference
***MYO7A***	c.1687G>A	p.(Gly563Ser)	Missense	Homozygous	USH1B	4	MYSc domain	Morocco	[16]
	c.653_658del	p.(Ile219_His220del)	In-frame deletion	Homozygous	USH1B	2	MYSc domain	Morocco	[9], this study
	c.2283-1G>T	p.?	Splice site	Homozygous	USH1B	1	IQ domain	Morocco	[9]
	c.6025delG	p.(Ala2009Profs*32)	Frameshift deletion	Compound heterozygous	DFNB2	3	B41 domain	Morocco	[17]
	c.6229T>A	p.(Trp2077Arg)	Missense				B41 domain	Morocco	
	c.3500T>A	p.(Leu1167His)	Missense	Compound heterozygous	DFNB2	8	MyTH4 domain	Morocco	
	c.5617C>T	p.(Arg1873Trp)	Missense				MyTH4 domain	Morocco	
	c.4487C>A	p.(Thr1496Lys)	Missense				Non-structural domain region	Morocco	
	c.1657 C>T	p.(His553Tyr)	Missense	Homozygous	USH1B	1	MYSc domain	Morocco	[18]
***USH1G***	c.742C>T	p.(Gln248*)	Nonsense	Homozygous	USH1G	1	Non-structural domain region	Not previously found in Moroccan population	This study
***WFS1***	c.1113G>A	p.(Trp371*)	Nonsense	Compound heterozygous	Wolfram syndrome	1	Loop region	Morocco	[19]
	c.1223_1224insGGAACCACCTGGAGCCCTATGCCCATTT	p.(Phe408Leufs*144)	Frameshift insertion				Transmembrane domain		
	c.1329C>G	p.(Ser443Arg)	Missense	Homozygous	Wolfram syndrome	2	Transmembrane domain	Morocco	[20]
	c.1113G>A	p.(Trp371*)	Nonsense	Homozygous	Wolfram syndrome	3	Loop region	Morocco	
	c.1235_1237del	p.(Phe414del)	In-frame deletion	Homozygous	Wolfram syndrome	1	Transmembrane domain	Not previously reported in Moroccan population	This study
***SMC1***	c.3186del	p.(Lys1063Argfs*149)	Frameshift deletion	Heterozygous	Rett-like syndrome	1	Coiled coil region 2	Morocco	[21]
	c.664C>T	p.(Gln222*)	Nonsense	Heterozygous	Cornelia de Lange syndrome	1	Coiled coil region 1	Morocco	
	c.2351T>C	p.(Ile784Thr)	Missense	Heterozygous	Cornelia de Lange syndrome, Rett-like syndrome	2	SMC hinge domain	Morocco	This study
***KCNQ1***	c.1343dupC	p.(Glu449Argfs*14)	Frameshift insertion	Homozygous	Jervell and Lange-Nielsen syndrome	1	Non-structural domain region	Morocco	[22]
	c.915G>A	p.(Trp305*)	Nonsense	Homozygous	Jervell and Lange-Nielsen syndrome	2	Transmembrane domain	Morocco	This study
***CHD7***	c.5405-2A>G	p.?	Splice site	Heterozygous	CHARGE syndrome	1	Non-structural domain region	Morocco/France/Belgium (unclear)	[23]
	c.5405-7G>A	p.?	Splice site	Heterozygous	CHARGE syndrome	12	Non-structural domain region	Morocco/France/Belgium (unclear)	
	c.5405-13G>A	p.?	Splice site	Heterozygous	CHARGE syndrome	1	Non-structural domain region	Morocco/France/Belgium (unclear)	
	c.5405-17G>A	p.?	Splice site	Heterozygous	CHARGE syndrome	7	Non-structural domain region	Morocco/France/Belgium (unclear)	
	c.5405-18C>A	p.?	Splice site	Heterozygous	CHARGE syndrome	2	Non-structural domain region	Morocco/France/Belgium (unclear)	
	~184 kb microdeletion	p.?	Microdeletion	NA	CHARGE syndrome	1	Non-structural domain region	Morocco	[24]
	c.5050G>A	p.(Gly1684Ser)	Missense	Heterozygous	CHARGE syndrome	1	Non-structural domain region	Morocco	This study
***MITF***	c.1031+1G>A	p.?	Splice site	Heterozygous	Waardenburg syndrome type 2A	1	Helix–loop–helix domain	Morocco	This study

**Table 3:** In silico pathogenicity predictions for missense and splicing variants.

Gene	Variant	Protein Change	CADD	AlphaMissense	MutationTaster	M-CAP	phyloP100way Vertebrate
*SMC1A*	c.2351T>C	p.(Ile784Thr)	25.1	Likely Pathogenic	Disease causing	Damaging	8.897
*CHD7*	c.5050G>A	p.(Gly1684Ser)	33	Likely Pathogenic	Disease causing	Damaging	10.003
**In silico** **pathogenicity predictions for splicing variant**							
**Gene**	**Variant**	**Donor loss score Δ**	**Acceptor loss score** **Δ**	**Pangolin splice loss** **score Δ**	**Pangolin splice gain** **score Δ**	**Dominant effect**	**Predicted interpretation**
*MITF*	c.1031+1G>A	0.99	0.60	0.82	0.34	Loss dominates	Strong disruption of canonical donor site; likely aberrant splicing

Δ values represent SpliceAI-predicted changes in splice donor or acceptor strength (gain or loss), ranging from 0 to 1.

## Data Availability

The data presented in this study are available upon request from the corresponding author. The data are not publicly available due to the confidentiality of patient information.

## Abbreviations

The following abbreviations are used in this manuscript:

ACMG	American College of Medical Genetics and Genomics
CADD	Combined Annotation-Dependent Depletion
CdLS	Cornelia de Lange Syndrome
CHARGE	Coloboma, Heart defects, Choanal atresia, Growth retardation, Genital anomalies, Ear abnormalities
DFNB2	Non-syndromic hearing loss
ENT	Ear, Nose, and Throat
ER	Endoplasmic Reticulum
ES	Exome Sequencing
Fam	Family
GATK	Genome Analysis Toolkit
gnomAD	Genome Aggregation Database
HL	Hearing Loss
HPO	Human Phenotype Ontology
JLNS	Jervell and Lange–Nielsen Syndrome
NSHL	Non-Syndromic Hearing Loss
SAMTools	Sequence Alignment/Map Tools
SHL	Syndromic Hearing Loss
SIFT	Sorting Intolerant From Tolerant
SNHL	Sensorineural Hearing Loss
USH	Usher Syndrome
USH1B	Usher Syndrome type 1B
USH1G	Usher Syndrome type 1G
VCF	Variant Call Format
WFS1	Wolfram Syndrome 1
WS1	Waardenburg Syndrome Type 1
WS2	Waardenburg Syndrome Type 2
